# Imaging assessment of a portable hemodialysis device: detection of possible failure modes and monitoring of functional performance

**DOI:** 10.7243/2052-6962-2-2

**Published:** 2014-03-27

**Authors:** Olufoladare G. Olorunsola, Steven H. Kim, Ryan Chang, Yuo-Chen Kuo, Steven W. Hetts, Alex Heller, Rishi Kant, Maythem Saeed, William H. Fissell, Shuvo Roy, Mark W. Wilson

**Affiliations:** 1 Department of Radiology and Biomedical Imaging, University of California, San Francisco, 505 Parnassus Ave., Room 381, Box 0628, San Francisco, CA 94143, USA; 2 Department of Medicine, Vanderbilt University Medical Center, USA

**Keywords:** End-stage renal disease, renal replacement therapy, hemodialysis device, multi-detector computed tomography

## Abstract

**Background:**

The purpose of this study was to investigate the utility and limitations of various imaging modalities in the noninvasive assessment of a novel compact hemodialyzer under development for renal replacement therapy, with specific aim towards monitoring its functional performance.

**Methods:**

The prototype is a 4×3×6 cm aluminum cartridge housing “blood” and “dialysate” flow paths arranged in parallel. A sheet of semipermeable silicon nanopore membranes forms the blood-dialysate interface, allowing passage of small molecules. Blood flow was simulated using a peristaltic pump to instill iodinated contrast through the blood compartment, while de-ionized water was instilled through the dialysate compartment at a matched rate in the countercurrent direction. Images were acquired under these flow conditions using multi-detector computed tomography (MDCT), fluoroscopy, high-resolution quantitative computed tomography (HR-QCT), and magnetic resonance imaging (MRI). MDCT was used to monitor contrast diffusion efficiency by plotting contrast density as a function of position along the path of flow through the cartridge during steady state infusion at 1 and 20 mL/min. Both linear and exponential regressions were used to model contrast decay along the flow path.

**Results:**

Both linear and exponential models of contrast decay appeared to be reasonable approximations, yielding similar results for contrast diffusion during a single pass through the cartridge. There was no measurable difference in contrast diffusion when comparing 1 mL/min and 20 mL/min flow rates. Fluoroscopy allowed a gross qualitative assessment of flow within the device, and revealed flow inhomogeneity within the corner of the cartridge opposite the blood inlet port. MRI and HR-QCT were both severely limited due to the paramagnetic properties and high atomic number of the target material, respectively. During testing, we encountered several causes of device malfunction, including leak formation, trapped gas, and contrast-mediated nanopore clogging. We illustrate the imaging manifestations of each.

**Conclusions:**

Despite the inherent challenges in imaging a predominantly metallic device, some modalities show potential in the non-invasive assessment of a novel compact hemodialyzer. The approaches described here could potentially be translated to device evaluation in the implanted setting.

## Introduction

End-stage renal disease (ESRD) is becoming increasingly common, in part due to the rising prevalence of hypertension and diabetes [1]. Kidney transplantation is a highly effective treatment, but remains limited due to the scarcity of donor organs. Therefore, the majority of patients are instead treated with thrice weekly in-center hemodialysis. Recent evidence associating more frequent and longer hemodialysis treatments with improved patient outcomes has stimulated efforts to increase the ease and availability of dialysis through alternative modalities [2–5]. One such venture is the bioartificial kidney project, in which investigators have incorporated advanced microlectromechanical systems membrane technology in a miniaturized dialyzer cartridge, able to accomplish successful hemofiltration of whole blood [6–9]. Establishing non-invasive means of evaluating such a device is crucial prior to use, and imaging will likely play a central role in this area. In this study, we investigate various imaging approaches to the preliminary evaluation of a prototype. Specifically, we aim to develop methods of monitoring functional performance. We also address the imaging appearances of several causes of device dysfunction.

## Methods

### Prototype design

**Figure 1:**
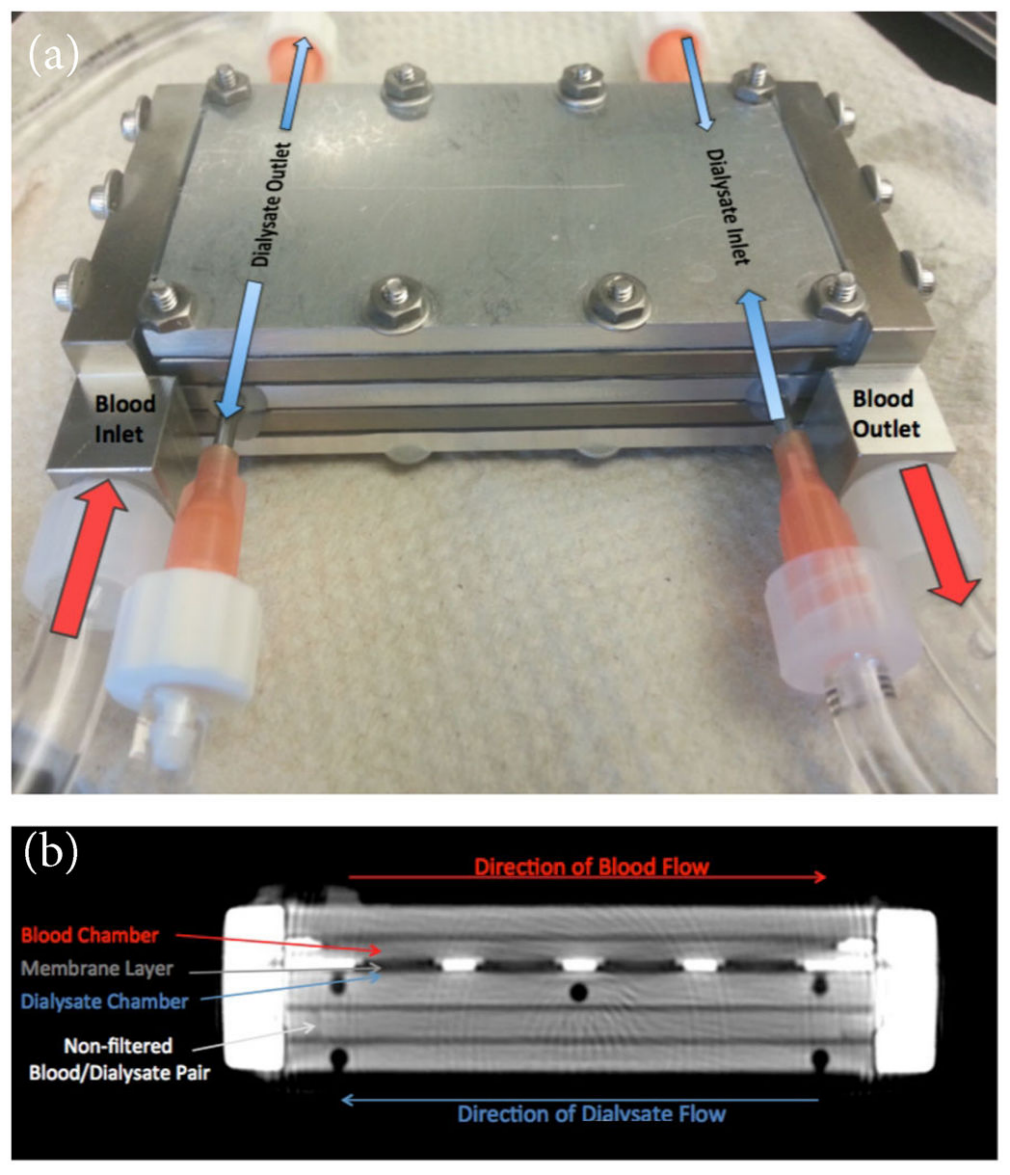
(**a**) Photograph of the portable hemodialysis device prototype showing ports on either end of the cartridge, which allow flow to be generated in countercurrent directions within the respective blood and dialysate chambers. (**b**) MDCT image depicting the internal structure, which consists of adjacent thin sheet-like chambers. In the superior half of the cartridge, a layer of filtration membranes separates neighboring blood and dialysate chambers. In the inferior half of the cartridge, neighboring blood and dialysate chambers are instead separated by a solid plate, which does not permit diffusion, thus establishing an internal control.

The prototype (Figure 1) consists of a 4×3×6 cm outer aluminum framework, housing a stack of four parallel sheet-like chambers. Two of these chambers comprise the ‘blood’ compartment, and two comprise the ‘dialysate’ compartment. The chambers are arranged such that each blood chamber is paired with its neighboring dialysate chamber, forming an interface with broad contact. One such blood-dialysate interface consists of a titanium mounting plate supporting eight silicon nanopore membranes (total effective surface area: 1.73 cm^2^; pore size: 7 nm×4 μm), which allow the diffusion of small solutes between the chambers. Membranes were polyethylene glycol (PEG) coated, which will be necessary for biocompatibility in future *in vivo* studies [10]. The other interface is composed of a ‘sham’ non-porous titanium plate, allowing zero diffusion, and thus serving as an internal control. Inflow and outflow ports positioned at either end of the cartridge enable flow to be generated within each chamber independently.

The device components were assembled using brass screws and aluminum washers, with silicone glue sealant. The membranes were fixed within the titanium mounting plate using a silicone-based organic polymer polydimethylsiloxane (PDMS). Device assembly and tubing connections and exchanges were performed during submersion in de-ionized water in attempt to exclude any air bubbles from within the device.

### Flow generation

Flow rates were equal and in opposite directions within the blood and dialysate compartments, respectively (countercurrent exchange). Iodinated contrast (Omnipaque [iohexol] 350; GE Healthcare, Inc., Princeton NJ) in various dilutions was instilled via the blood ports, while de-ionized water was instilled via the dialysate ports. Flow was powered using a peristaltic pump (Master Flex L/S, Cole-Parmer, Vernon Hills, IL), coupled to size LS-14 Precision silicone tubing (Cole- Parmer, Vernon Hills, IL). Flow rates were validated on-site to within 5% accuracy. All specified rates refer to flow within each compartment.

### Qualitative flow assessment

X-ray fluoroscopy was performed using the Innova 4100 Angiographic Imaging System (GE Healthcare, Waukesha, WI). Following a pre-contrast spot image, cine images were acquired in two projections during a 90-second dynamic infusion of Omnipaque 350 via the blood inlet port at a rate of 20 mL/min. Sequences were viewed using a PACS workstation to qualitatively assess flow patterns.

### Quantitative diffusion data acquisition and analysis

Diffusion data were obtained using a 64-detector MDCT scanner (Light Speed, GE Medical Systems, Milwaukee, WI). Images were helically acquired at 140 kV and 250mA. The image plane of acquisition was chosen such that the flow paths were displayed in cross-section (imaging plane oriented orthogonal to the chamber orientation and parallel to the direction of flow, as in Figure 1b). Data were reconstructed at 0.625 mm slice thickness in both bone and soft tissue algorithms, and reformations in orthogonal planes were generated using a Tera Recon Advanced 3D workstation (Tera Recon, Inc., San Mateo, CA).

**Figure 2:**
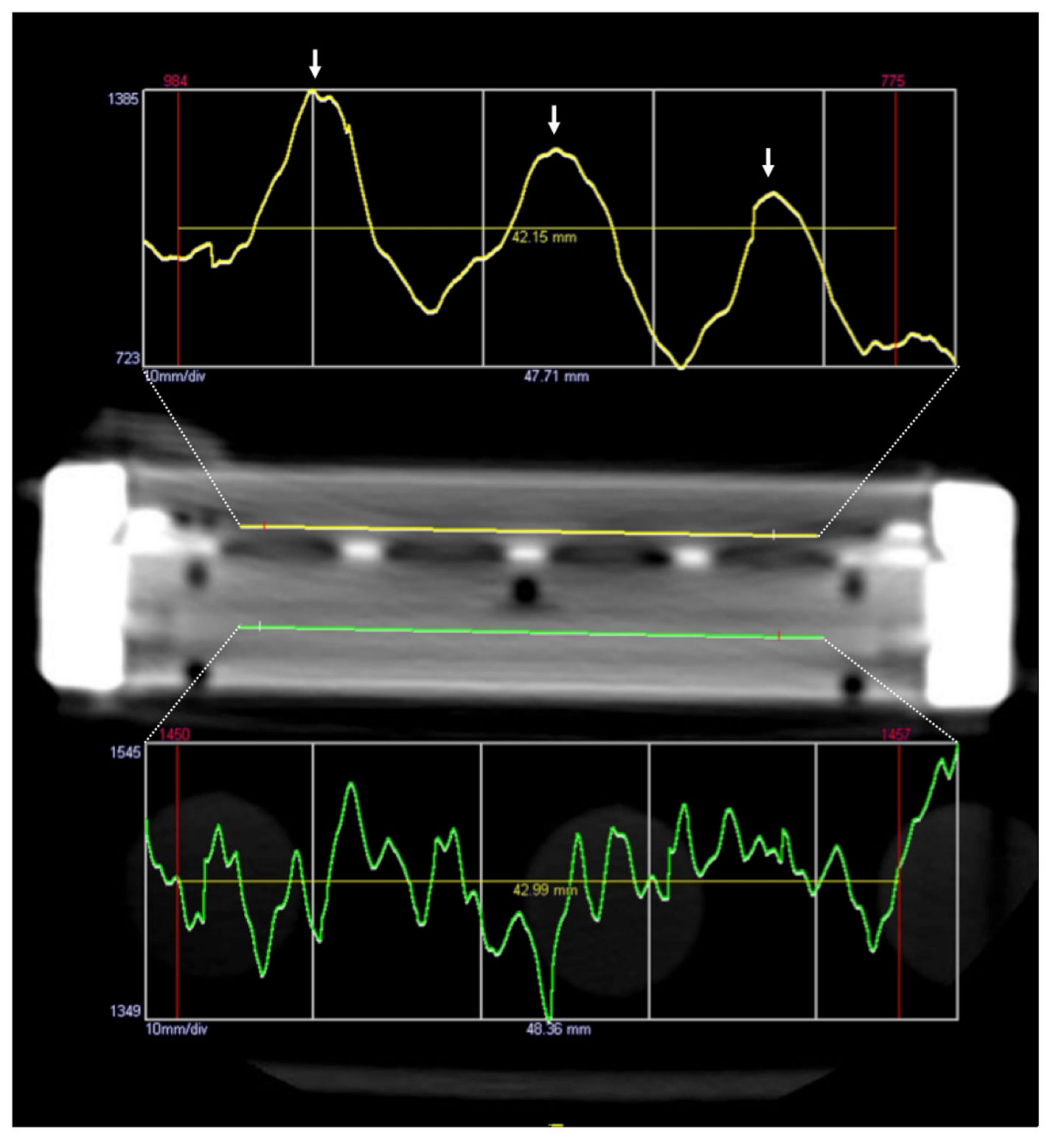
A density profile (*yellow line*) drawn along the length of the superior blood chamber, which is subject to diffusion, shows a decline in HU in the direction of blood flow from left to right. Superimposed periodic peaks (*white arrows*) represent partial volume averaging from the intervening metal struts supporting the membranes. As we would expect, the inferior blood chamber, which is not subject to diffusion, shows a flat density profile (*green line*) and serves as an internal control.

To quantify contrast diffusion by MDCT, we used density in Hounsfield units (HU) as a proxy for contrast concentration, taking advantage of the direct linear relationship between the two variables [11]. We plotted density versus position along the length of the cartridge in the direction of contrast flow. This was accomplished by drawing a density profile within the blood compartment along the longitudinal axis of the cartridge using a Tera Recon Advanced 3D workstation (Figure 2). Both linear and exponential regressions were fit to the data. The comparable measurement was applied to the pre-contrast series in each experiment, to serve as the baseline (intrinsic density of the water-filled cartridge). A linear function was fit to the pre-contrast data, which was then subtracted from the post-contrast linear and exponential regressions.

**Figure 3:**
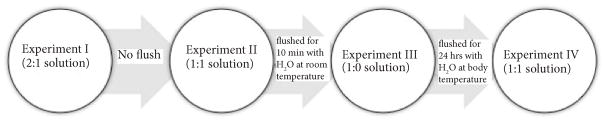
Experimental sequence I through IV showing progressive escalation of the cartridge flushing regimen. The strength of the contrast solution used in each experiment is noted in parentheses (Omnipaque 350: de-ionized water).

A sequence of four MDCT diffusion experiments (designated I through IV) was performed to investigate the hypothesis that clogging of the membrane pores may occur at high contrast concentrations. Each sequential experiment consisted of a non-contrast acquisition followed by two post-contrast acquisitions at steady state flow rates of 1 mL/min (minimum deliverable rate) and 20 mL/min (maximum rate not exceeding an estimated pressure of 1 psi within the system), respectively. All studies were conducted at room temperature (21°C) using iodinated contrast solutions ranging in strength from pure Omnipaque 350 to a 1:1 solution of Omnipaque 350 and deionized water. Intensity of cartridge flushing was progressively escalated between experiments, ranging from no flushing between Experiments I and II, to flushing for 24 hours at 38°C between experiments III and IV, as depicted in (Figure 3).

### Other imaging modalities

#### MRI

MR imaging was attempted to determine feasibility on a 3-Tesla GE 15.4 MRI (GE Healthcare). The device was positioned within a head coil and surrounded by saline bags, while standard T2 FSE and T1 spin-echo sequences were acquired.

#### High-resolution quantitative computed tomography (HR-QCT)

Imaging of the entire cartridge was attempted to determine feasibility using the Xtreme CT HR-pQCT (Scanco Medical AG; Brüttisellen, Switzerland). Images were acquired at the maximum attainable tube voltage of 60 kV.

#### Scanning electron microscopy (SEM)

Under SEM, we examined both a naive membrane and a contrast-exposed membrane (harvested from the disassembled device following experimental sequence I through IV). Specimens were attached to aluminum specimen blocks with adhesive carbon tabs, sputter coated with Palladium-Gold, and examined in a Nikon/JEOL Neoscope SEM. An accelerating voltage of 10 or 15 kV was applied and the secondary electron signal was recorded. A second observation was made after the samples were attached at a 45-degree angle with colloidal silver paste.

## Results

**Figure 4:**
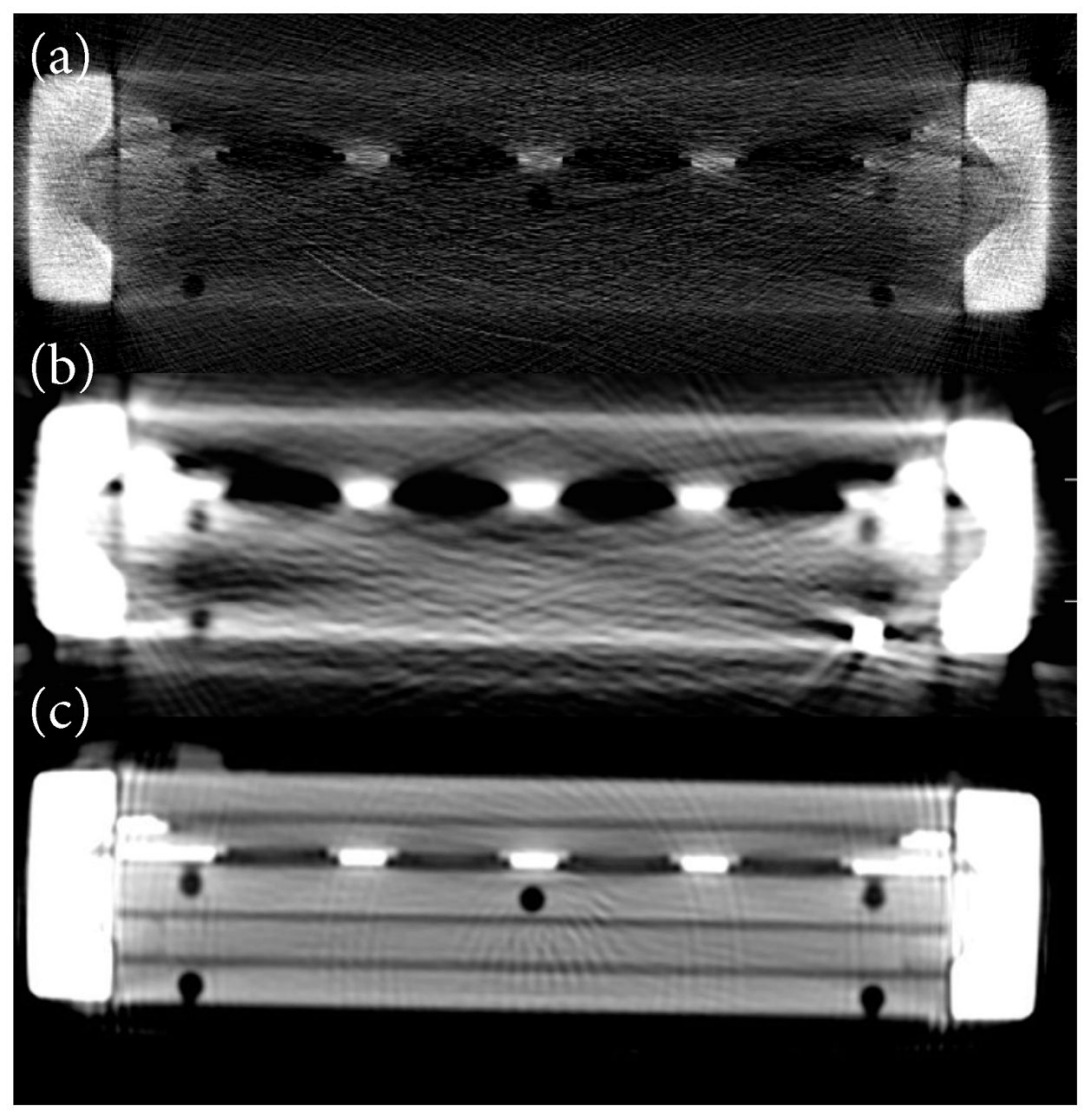
HR-QCT image acquired at 60 kV (**a**), and conventional MDCT images acquired at 80 kV (**b**) and 140 kV (**c**). As tube potential was increased, image quality improved due to greater penetration and decreased scattering of photons, resulting in an improved signal-to-noise ratio.

We encountered two major limitations to MDCT imaging. For one, image quality suffered from beam-hardening and photon starvation due to the high x-ray attenuating properties of the metallic device components. There was significant improvement in image quality with increasing tube voltage, ranging between 60 and 140 kV (Figure 4). Another limitation stemmed from inhomogeneity of the filtration plate, which consisted of low-density membrane units alternating with the intervening metallic-density supporting plate struts. The periodically oscillating density was artefactually detected in the adjacent blood compartment (region of interest) due to partial volume averaging. This effect was evident in the contrast density profiles (Figure 2).

**Figure 5:**
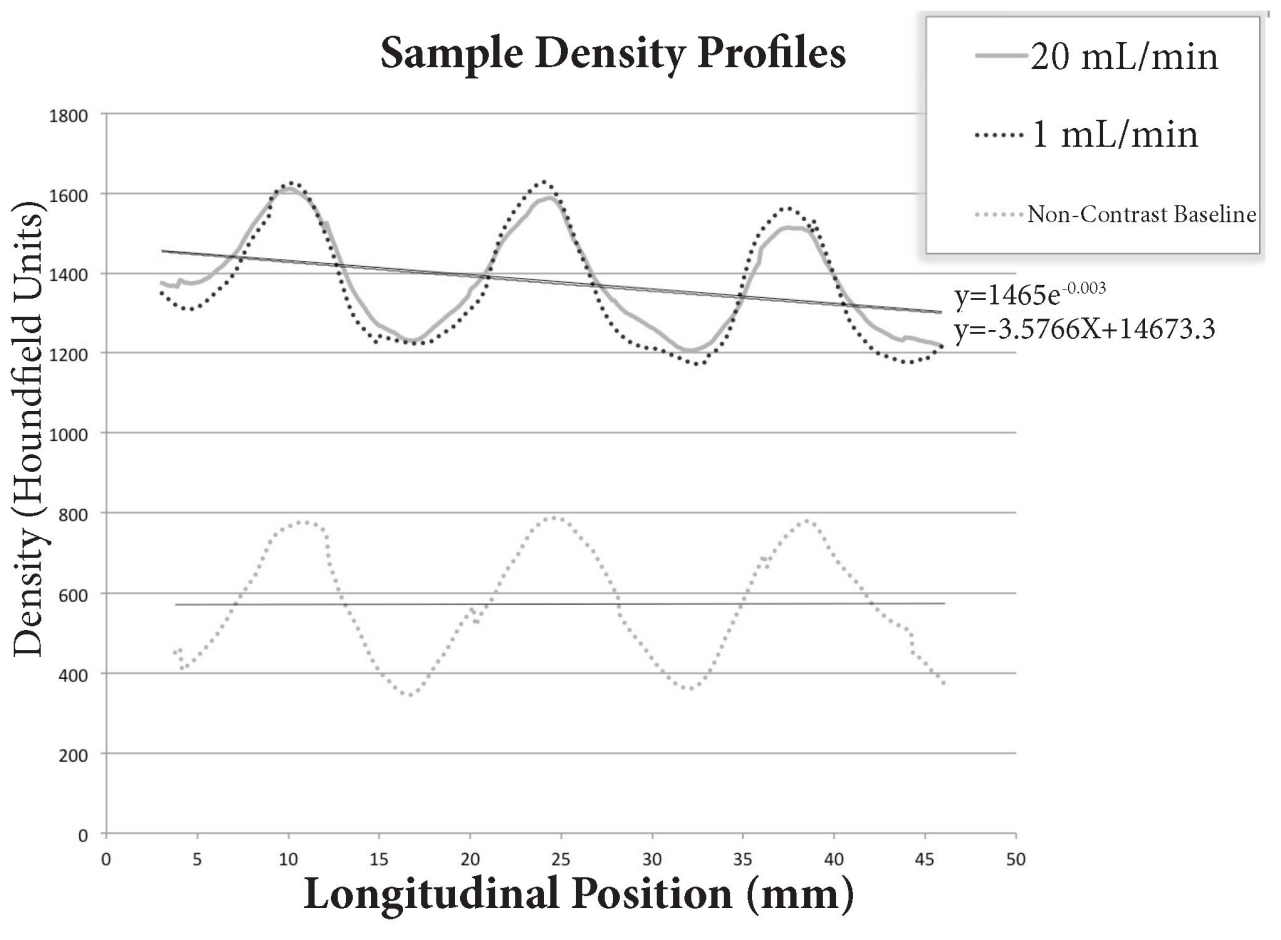
Sample density profiles showing contrast decay along the length of the cartridge at 1 and 20 mL/min flow rates. Both exponential and linear regressions are shown. The pre-contrast baseline data are also included.

Despite its limitations, MDCT provided a semi-quantitative assessment of contrast diffusion by measuring the decrement in contrast density along the flow path through the cartridge (Figure 5). Diffusion estimates are reported as ‘single-pass diffusion’, defined as the percent decrease in density from the proximal to distal end of the blood compartment, after correcting for the pre-contrast baseline. Both linear and exponential approximations yielded similar values for diffusion, with exponential regressions overall showing slightly better R2 values compared to linear regressions.

**Figure 6:**
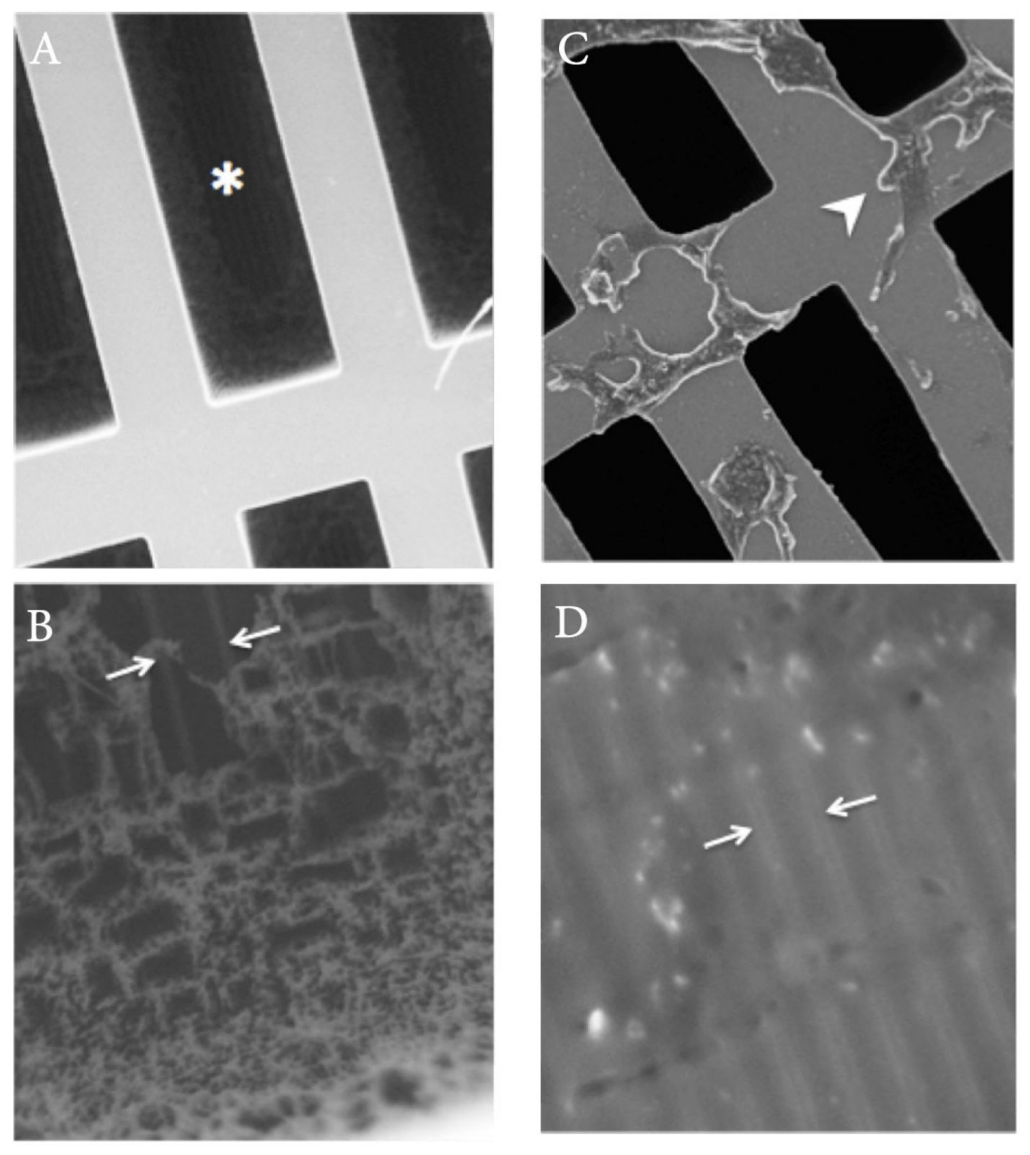
SEM at 300X (**a**) and 2200X (**b**) of a naive membrane prior to contrast exposure, demonstrating the silicon framework harboring numerous slit-like windows (*asterisk*), each of which contains a fine network of pores (*bracketed by arrows*). In distinction, a contrast-exposed membrane at 500X (**c**) and 10000X (**d**) demonstrates patchy areas of amorphous semi-translucent material thickly coating the silicon framework (*arrowhea*d) and completely sealing and obstructing the pores (*bracketed by arrows*) in many areas.

Estimated diffusion values for Experiments I through IV are summarized in Table 1. Flow rates of 1 mL/min and 20 mL/min showed similar single-pass diffusion in each experiment. A serial decline in single-pass diffusion was observed between Experiments I and II and between Experiments II and III, associated with no or mild intervening flushing. More aggressive membrane flushing using heated de-ionized water between Experiments III and IV was associated with a partial recovery of diffusion efficiency. An explanted membrane (following Experiments I through IV) was observed under SEM to evaluate for pore clogging. This demonstrated patchy areas of amorphous material thickly coating the membranes and completely covering the pores in many areas, whereas a naive membrane showed patent pores (Figure 6).

**Figure 7:**
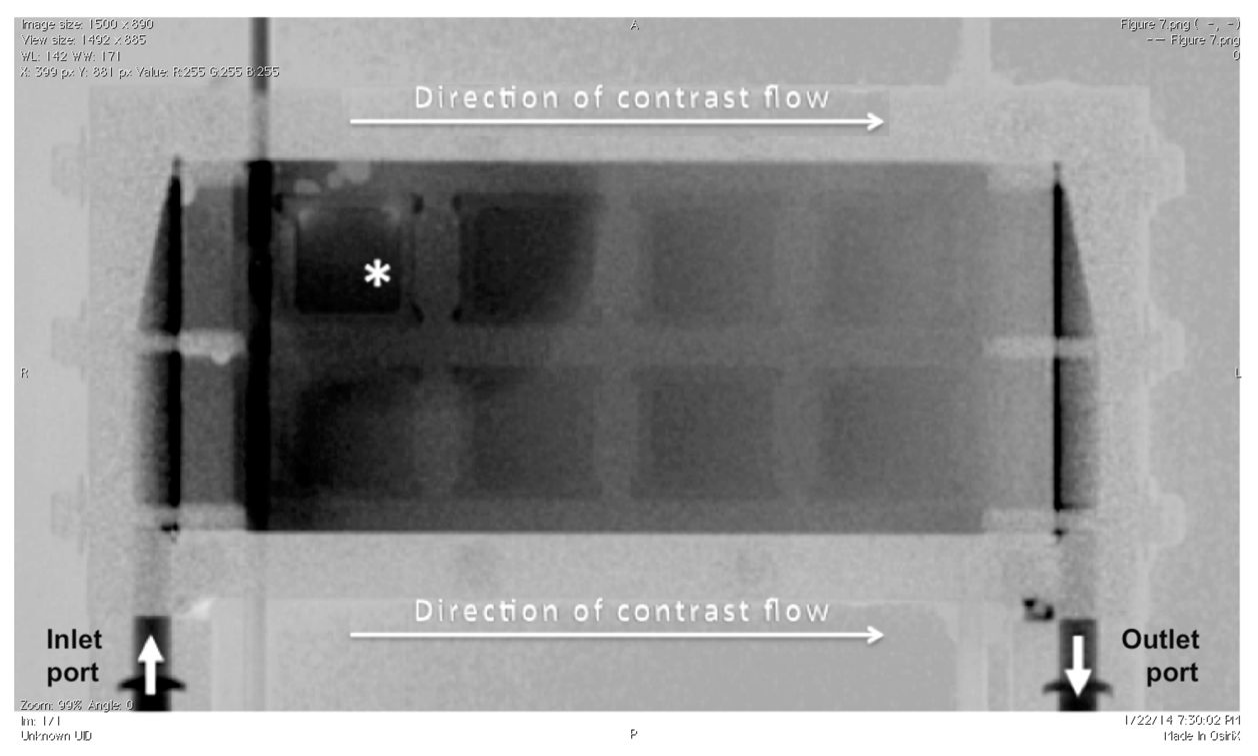
Digitally subtracted fluoroscopic image (top down view) obtained during initiation of flow within the cartridge. Arrows indicate the direction of flow. Regionally higher contrast density is observed across from the inlet port (*asterisk*), suggesting recirculation resulting in preferential accumulation of contrast in this area.

Fluoroscopy provided a gross visual assessment of contrast flow within the device. Cine images demonstrated flow inhomogeneity, with early preferential accumulation of contrast in the corner of the device across from the inlet port, suggesting recirculation in this region (Figure 7). As would be expected, fluoroscopy proved less susceptible to degradation by metallic artifact compared to tomographic imaging. The other imaging modalities assayed (MRI and HR-QCT) were limited by the device material. For example, MR imaging of the cartridge at 3 Tesla initially generated no detectable signal. After surrounding the device with saline bags, it was evident that the fixation screws (composed of brass and aluminum) were contributing to confluent metallic susceptibility artifact, obscuring signal from any of the fluid containing spaces within the device. HR-QCT imaging of the cartridge was limited by low maximum tube voltage (60 kV). These low-energy photons demonstrated very limited ability to penetrate the high-atomic number material forming the outer shell of the device.

### Failure modes

On several occasions, a cause of device malfunction was depicted radiographically, either confirming a suspected abnormality or representing an incidental finding. Several examples include the following.

#### Trapped gas

**Figure 8:**
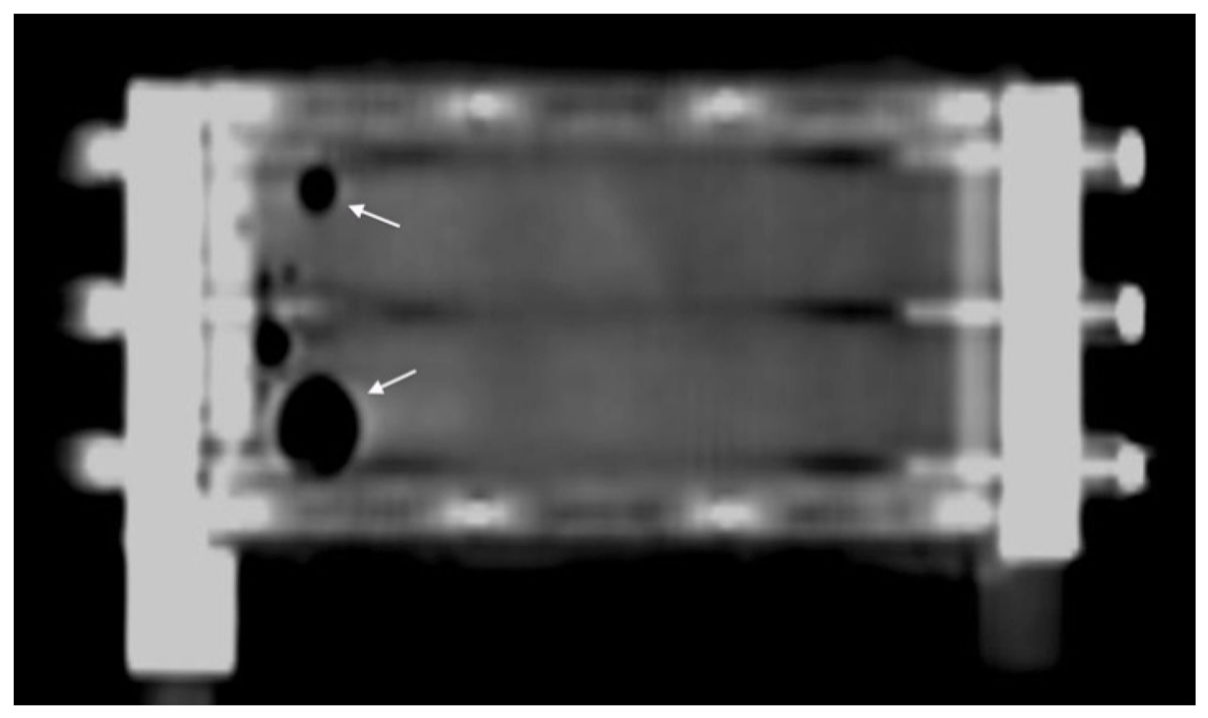
MDCT image reformatted in the plane of the blood chamber, which is filled with contrast. Trapped locules of gas can be seen as air-density filling defects outlined by contrast within the blood chamber (*arrows*).

To varying degrees, small pockets of gas were evident during imaging, either within the blood compartment, dialysate compartment, or both (Figure 8). The locules tended to lodge along seams at the membrane-titanium plate interfaces due to surface tension, and demonstrated substantial resistance to expulsion. Purging of the gas was best accomplished by gentle vibration combined with brisk flow.

#### External Leak

**Figure 9:**
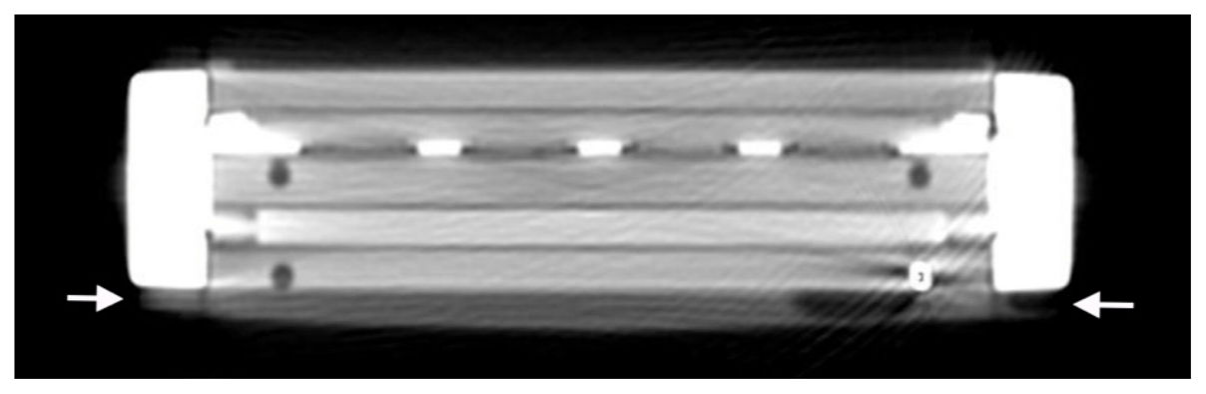
MDCT image during contrast instillation showing puddling of dense material below the cartridge (*bracketed by arrows*), which progressively increased in density over serial time points, indicating active extravasation of contrast from the blood compartment.

Following initial device assembly, MDCT imaging during instillation of non-dilute contrast demonstrated dense fluid puddling beneath the cartridge, which progressively increased in density over serial acquisitions (Figure 9). This finding represented active leaking from the blood compartment. The leak was subsequently repaired by reinforcing modular junctions using excess silicone glue.

#### Internal (Inter-compartmental) Leak

**Figure 10:**
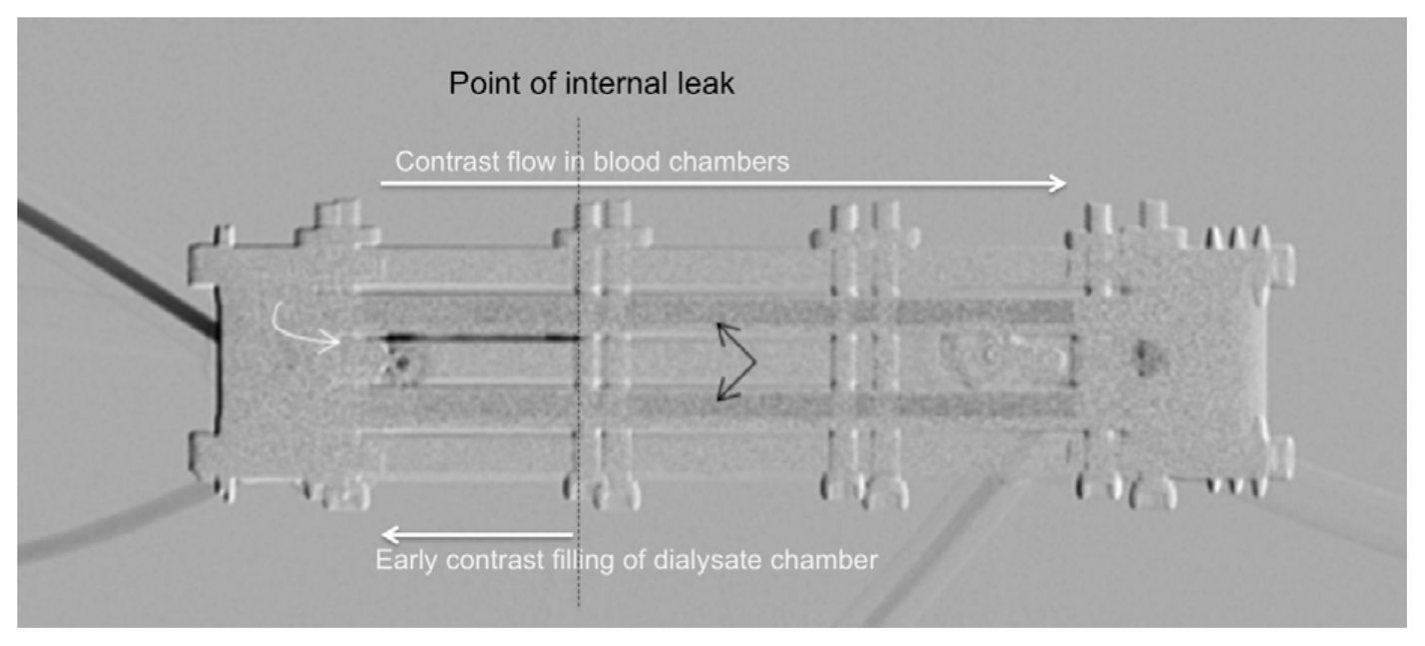
Digitally subtracted fluoroscopic image obtained during contrast instillation via the blood port. The two layers of fainter density (*black arrows*) represent contrast opacifying the superior and inferior blood chambers. The linear very high density (*curved white arrow*) represents unexpectedly early filling of the dialysate chamber with contrast, which is too dense to be the result of diffusion. This instead represents an intercompartmental leak from the adjacent blood compartment, with localization of the leak indicated by the dashed black line.

On several occasions, a communication developed between the blood and dialysate compartments. As with the external leak, this occurred using non-dilute contrast solution. Based on our experience with the device, it appears that a rapid change in pressure within the cartridge (as occurs with abrupt initiation of flow) may result in fracture of the membranes units. This problem was best shown by fluoroscopy, which demonstrated immediate filling of the dialysate chamber with contrast instilled via the blood compartment. This occurred too briskly and was too dense to be the result of contrast diffusion. The communication appeared localized to a single point near the proximal end of the cartridge (Figure 10). An inter-compartmental leak was confirmed upon disassembly of the device, revealing a fractured and loosened membrane, corresponding in location to the imaging abnormality. The damaged membrane unit was subsequently replaced, with return of normal device function.

#### Component fracture

**Figure 11:**
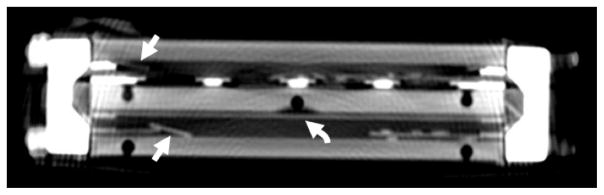
MDCT image without contrast demonstrating comminuted full-thickness fractures of multiple silicon plates (*straight arrows*). Numerous displaced fragments are scattered within the cartridge. One silicon plate remains intact (*curved arrow*). This internal derangement was heralded by a large internal leak.

On one occasion, forceful impact to the device resulted in a new large communication between the blood and dialysate compartments, manifesting as coupling of flow between compartments (flow generated within the blood compartment was transmitted to the dialysate compartment, and vice versa). Based on this behavior, major damage to the internal structure of the device was suspected, and a non-contrast CT was obtained for further evaluation. This demonstrated comminuted full-thickness fractures of the internal silicon plates, with numerous displaced fragments (Figure 11). The device was disassembled and correlation was made with visual inspection, confirming complex fractures of two of the silicon plates resulting in wide communication between neighboring blood and dialysate compartments.

#### Intrinsic membrane dysfunction

As described above, exposure to concentrated iodinated contrast material resulted in clogging of the membrane nanopores, with an associated decline in function.

## Discussion

The present study was motivated by the anticipated need to develop non-invasive means for assessing a novel portable hemodialysis device. We investigated various imaging modalities in this capacity, disclosing various device failure modes in the process. The most promising modality was MDCT, which not only provided detailed structural information, but also allowed a functional assessment with regard to membrane diffusion. The methods described here could prove valuable in the implanted setting for monitoring device function.

### Failure modes

**Figure 12:**
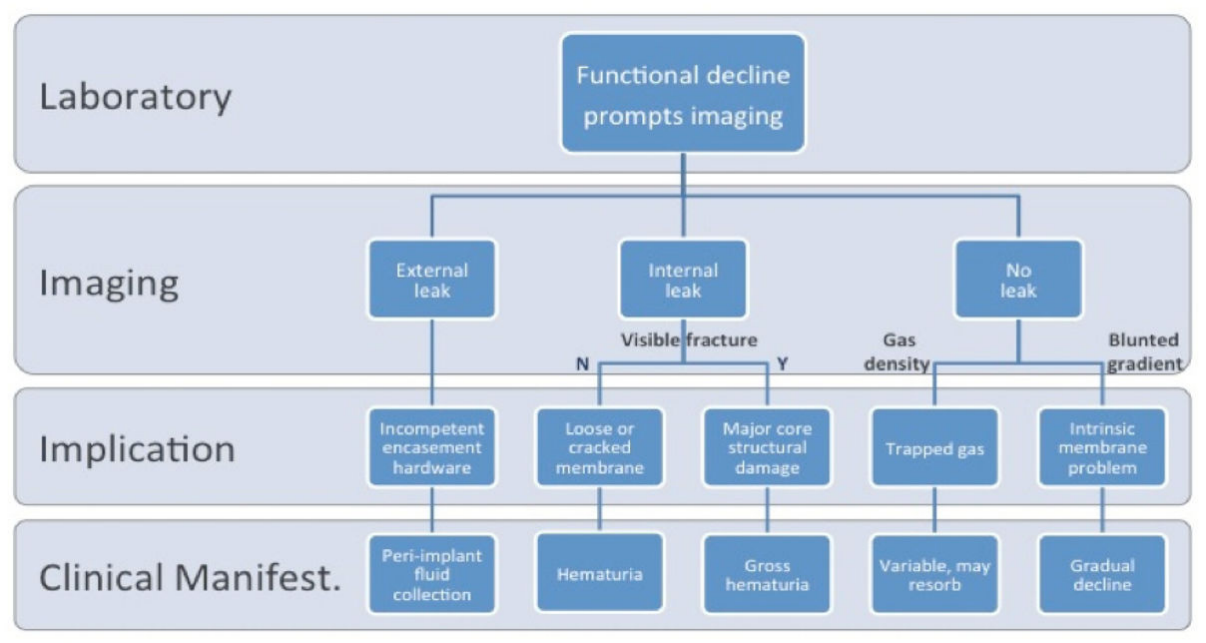
Outline of various failure modes, relating imaging findings to clinical manifestations as might be encountered in the implanted setting.

Imaging is well established in detecting certain complications related to medical implants. For example, fluoroscopy and CT have demonstrated leaks from intrathecal medication pumps [12]. Similarly, several reports describe the detection of thrombus related to left ventricular assist devices using CT [13–15]. In the present study of a portable hemodialysis prototype, we encountered several failure modes, including trapped gas, structural incompetency resulting in internal or external leaks, and fractured components. Additionally, we observed declining membrane function in the absence of any of the above causes, associated with contrast-mediated clogging of the membrane nanopores shown by SEM. Any of these failure modes, if encountered in the implanted setting, would likely manifest clinically as a decline in function. (Figure 12) predicts how such imaging findings might eventually play a role in non-invasively evaluating clinically suspected device dysfunction in the implanted setting.

It is worth noting that leakage may be related to the viscosity of the fluid used. We frequently used 1:1 dilute Omnipaque 350 at room temperature, the viscosity of which is approximately 3.1 cp, closely approximating that of whole blood at body temperature [16].

### Diffusion

It is unclear how to best model the contrast diffusion kinetics from our MDCT data. Thus, we applied both linear and exponential regression models, recognizing that both represent imperfect approximations. Given this uncertainty, our results should not be interpreted as an exact quantification of diffusion, but rather as relative values illustrating trends in diffusion over time. For example, we were able to detect decreasing diffusion associated with contrast-mediated membrane pore clogging.

There was no penalty in terms of single-pass diffusion at 20 mL/min compared to 1 mL/min, suggesting rapid diffusion across the membranes, which is not rate-limited in this range of flow rates. Importantly, our results pertain to the small uncharged molecule iohexol (821 Daltons), and are likely not generalizable to solutes with different properties. Diffusivity of iohexol cannot be determined, as clearance was not calculated in this study.

### Flow

A qualitative fluoroscopic assessment of contrast flow within the device revealed early preferential accumulation of contrast in the corner of the device across from the contrast inlet port, suggesting recirculation. If future device modifications were to achieve MRI compatibility, quantitative flow mapping and flow velocity measurements might be possible [17]. This type of assessment is important because inhomogeneous flow might not only reduce actual diffusion efficiency relative to the ideal, but may also increase predispose to intra-device thrombus formation in the implanted setting. Although not evaluated in the present study, MDCT could prove useful in depicting thrombus within the device, which would likely manifest as filling defects. The feasibility of MDCT for this purpose would need to be evaluated in future studies using blood products.

## Limitations

This study has several limitations. For one, there were two interrelated variables in experimental sequence I through IV: contrast concentration and degree of cartridge flushing. With the present study design, it is not possible to separate their effects on the observed pore clogging, with both variables likely playing a role. Notably, our studies sed supra-physiologic contrast concentrations at room temperature. Further studies are necessary to determine to what degree, if any, pore clogging would be a factor using lower concentrations of contrast at body temperature, since it is known that the viscosity of iohexol varies linearly with temperature and quadratically with concentration [18].

Imaging a predominantly metal device presented several inherent challenges. MRI, a potentially powerful tool in measuring flow, was ineffective due to overwhelming metallic susceptibility artifact. The device composition also posed a substantial challenge to imaging by MDCT, with the highly attenuating metallic components resulting in severe beam-hardening, limiting the sensitivity for the detection of subtle contrast differences. Density profiles were further degraded by superimposed periodic peaks representing partial volume averaging from the adjacent membrane units alternating with intervening metal struts, introducing error in our calculated density gradients. In order to overcome these factors, we used high contrast concentrations, which in turn promoted pore clogging.

Image quality was significantly improved by increasing tube voltage to 140 kV, the maximum capability of our conventional MDCT scanner, which suggests a possible role for future studies using megavoltage CT. One of numerous metal artifact suppression algorithms might also be considered, although could result in data distortion in ways that are difficult to predict, and would therefore require validation with an existing imaging model. Imaging quality will likely be most improved by varying the constituent materials of the device within structural constraints.

## Conclusions

In summary, this study outlines our initial experience using various imaging approaches in the evaluation of a portable hemodialysis device. Despite the inherent challenges in imaging a predominantly metallic device, several modalities show potential for non-invasive structural and functional assessment. The approaches described here could potentially be translated to device evaluation in the implanted setting.

## Figures and Tables

**Table 1:** Linear and exponential models of density decay along the length of the cartridge (after correcting for the pre-contrast density), used to estimate single-pass filtration.

		Linear Decay Model				Expon. Decay Model			

	Flow Rate (mL/min)	Intercept	Slope	% 1-Pass Filtration	R^2^	Intercept	Decay Constant	% 1-Pass Filtration	R^2^
**Exp. #1**	1	976^a^	−7.45	36.6	0.25	1013	−0.01	38.1	0.30
	20	1136	−7.65	32.3	0.35	1106	−0.009	35.1	0.40

**Exp. #2**	1	716	−4.31	28.9	0.12	703	−0.008	31.9	0.14
	20	747	−4.91	31.6	0.18	754	−0.009	35.1	0.21

**Exp. #3**	1	1491	−1.63	5.2	0.11	1487	−0.002	9.2	0.11
	20	1498	−1.74	5.6	0.16	1497	−0.002	9.2	0.16

**Exp. #4**	1	893	−3.43	18.4	0.09	889	−0.004	17.5	0.10
	20	928	−4.21	21.8	0.18	928	−0.005	21.3	0.20

a Values are baseline-corrected using the pre-contrast density.
